# Quill-Dwelling Cheyletid Mites in Birds of Prey: A New Genus from *Haliaeetus albicilla* and Evidence of Feather-Shaft Modification †

**DOI:** 10.3390/ani16193151

**Published:** 2026-10-08

**Authors:** Katarzyna Kaszewska-Gilas, Olaf Gwiazdowski, Tadeusz Mizera, Eliza Głowska-Patyniak

**Affiliations:** 1 Department of Animal Morphology, Faculty of Biology, Adam Mickiewicz University, Poznań, ul. Uniwersytetu Poznańskiego 6, 61-614 Poznań, Poland; olagwi@st.amu.edu.pl; 2 Department of Zoology, Poznań University of Life Sciences, ul. Wojska Polskiego 71C, 60-625 Poznań, Poland; tadeusz.mizera@up.poznan.pl

**Keywords:** Cheyletoidea, Metacheyletinii, *Aetocheyletia*, birds of prey, feather quill, setae anomalies

## Abstract

A new genus and species, *Aetocheyletia variata* gen n., sp. n. (Cheyletoidea: Cheyletidae), is described from the White-Tailed Eagle *Haliaeetus albicilla* (Linnaeus) (Accipitriformes: Accipitridae) in Poland. This represents the first record of cheyletid mites of the tribe Metacheyletinii from birds of prey. The new genus is morphologically most similar to *Metacheyletia* Fain, 1972, but differs from it in the presence of subcapitular setae *n* and an additional minute ventral tooth on the palpal claw. We further document considerable variability in body chaetotaxy observed in nearly all examined females. The most frequent anomalies occur in the aggenital and genito-anal regions and include (i) duplication of setae arising from a single alveolus, (ii) supernumerary setae, and (iii) reduction. In addition, distinct oval, circular, and spiral deformations of the quill wall were observed exclusively in specimens infested by *A*. *variata*, occurring in six of these feathers. No comparable alterations were found among the remaining five, including those hosting other mite taxa. This distribution suggests an association within the examined material, although causality remains unresolved. The present study expands current knowledge of the diversity and host range of Metacheyletinii and provides new observations on morphological variability and potential interactions between quill-dwelling mites and feather structure.

## Simple Summary

This study describes a new genus and species of mite, *Aetocheyletia variata* gen n., sp. n., found on the White-Tailed Eagle in Poland. It is the first known representative of this mite group recorded from birds of prey. The mites showed unusual variation in body setae, including extra, duplicated, or missing setae. Feather deformities were found only in feathers infested by these mites, suggesting that the infestation may be linked to feather damage. This discovery expands current knowledge of mite diversity, host associations, and the possible effects of these parasites on birds.

## 1. Introduction

Within the diverse assemblage of mites associated with birds, prostigmatic mites (Acariformes: Prostigmata) are one of the most species-rich and evolutionarily advanced groups. Avian-associated Prostigmata are highly specialized organisms inhabiting a wide range of microhabitats, including feather quills, skin, intracutaneous layers, and the respiratory tract [1]. These mites belong to several families grouped within three major superfamilies: Cheyletoidea (Cheyletidae, Harpirhynchidae, and Syringophilidae), Cloacaroidea (Cloacaridae), and Tydeoidea (Ereynetidae) [1,2]. Within Cheyletidae, three tribes are currently recognized: Cheletosomatini, comprising mainly predatory mites; Ornithocheyletiini, associated with the skin of birds; and Metacheyletinii, which inhabit feather quills [1,3]. The latter group remains particularly poorly known in terms of both taxonomy and biology.

The tribe Metacheyletinii is presently represented by a single genus, *Metacheyletia* Fain, 1972 [4], which includes a small number of described species. Until now, five species are recognized. *Metacheyletia degenerata* Fain and Bochkov, 2003, has been reported from passerine hosts, *Serinus mozambicus* (Fringillidae) [5], as well as from the coraciiform host *Merops apiaster* (Meropidae) [6]. *M*. *ngaii* Bochkov and Skoracki, 2011, was collected from the turaco *Corythaixoides leucogaster* (Musophagiformes: Musophagidae) [7]. Three further species parasitize parrots (Psittaculidae): *M*. *obesa* Fain, 1972, associated with *Psittacula krameria* [4,8], *M*. *longisetosa* Atyeo, Kethley and Perez, 1984, from *Amazona finschi* [9], and *M*. *amazonae* Bochkov and OConnor, 2003, from *Amazona amazonica* [10]. Members of this genus are characterized by a strongly reduced, larviform morphology, including the retention of only three pairs of functional legs and a pronounced reduction in idiosomal chaetotaxy [8,9]. Despite these distinctive features, Metacheyletinii remain one of the least studied lineages of bird-associated mites. Current knowledge is limited to isolated taxonomic descriptions, with very little information on their diversity, host specificity, geographic distribution, and ecological interactions. In particular, birds of prey (Accipitriformes) have not previously been reported as hosts of this group, which may reflect either true host specificity or, more likely, insufficient sampling of these hosts.

In addition, morphological variability within Metacheyletinii has received little attention. While reduction in body structures is a common feature of quill-inhabiting mites, the extent to which this reduction is accompanied by instability in characters such as chaetotaxy has not been systematically investigated. As a result, the occurrence and significance of anomalies in setation patterns remain largely unexplored in this group.

Another major gap concerns the potential impact of quill-dwelling cheyletids on feather structure. Although mites inhabiting feather quills are known to exploit a protected and relatively stable microhabitat, their interactions with feather tissues remain poorly understood. Reports of feather damage or structural alterations associated with mites are scarce and largely anecdotal [1], and the mechanisms underlying such changes, whether mechanical, developmental, or mediated by secondary factors, remain unresolved.

In this study, we describe a new genus and species of Metacheyletinii, *Aetocheyletia variata* gen. n., sp. n., collected from the body feathers and tail coverts of the White-Tailed Eagle, *Haliaeetus albicilla* (Accipitriformes: Accipitridae), in Poland. This finding represents the first record of cheyletid mites of this tribe from birds of prey and expands the known host range of the group. Furthermore, we document localized variability in idiosomal chaetotaxy among the examined females and report previously undescribed deformations of feather quills occurring in association with infestation by this species. By integrating taxonomic, morphological, and observational evidence, this study contributes to filling several gaps in current knowledge by (i) expanding the diversity and host associations of Metacheyletinii, (ii) providing new data on intraspecific morphological variability, and (iii) offering novel observations on potential interactions between quill-dwelling mites and feather microstructure. These results highlight the need for further integrative studies on the biology and ecological significance of quill-inhabiting mites.

## 2. Materials and Methods

### 2.1. Material Examined

In total, five individuals of the White-Tailed Eagle (*Haliaeetus albicilla*; Accipitriformes: Accipitridae) were examined for the presence of quill mites. Feather samples were obtained from following sources: (1) a deceased individual (suspected poisoning) found in Karwin, Lubuskie Voivodeship, Poland, on 26 December 2023 (coll. Tadeusz Mizera); (2) naturally moulted feathers collected in the field in Biały Bór, Zachodniopomorskie Voivodeship, Poland, on 12 August 2024 (coll. Ignacy Gołębiewski); (3) a deceased adult found after a collision with a wind turbine in Rozbitek, Wielkopolskie Voivodeship, Poland, collected on 23 September 2024 (coll. Tadeusz Mizera); (4) a deceased individual found in the Bolewice Forest District, Wielkopolskie Voivodeship, Poland, transported to the Wildlife Rehabilitation Centre in Spytkówki, Wielkopolskie Voivodeship, operated by the Z Dzikiej Strony Foundation, where it died in February 2025 (coll. Paulina Cudna); and (5) a deceased adult (suspected poisoning) found in Śmigiel, Wielkopolskie Voivodeship, Poland, on 4 March 2025 (coll. Paulina Cudna). Feathers representing different plumage types were initially examined under a Leica stereomicroscope to detect the presence of mites within the quills. Because feather sampling depended on the material available from each specimen (whole carcasses, moulted feathers, or material delivered to a rehabilitation centre), the total number of feathers screened was not systematically recorded across individuals. Screening continued until representative coverage of the major plumage tracts was achieved for each bird. Approximately 30 bodies, tail, and wings coverts were sampled per examined microhabitat, while all remiges and rectrices were collected. Quills containing mites were subsequently opened using a fine scalpel and examined in detail under a dissecting microscope. For each collected feather, we recorded the presence or absence of *Aetocheyletia*, the presence of other mite taxa, and the occurrence of quill-wall deformations. Mite-infected feathers (collected from two birds) were analyzed in the taxonomic laboratory at the Department of Animal Morphology, Faculty of Biology, Adam Mickiewicz University in Poznań.

In total, 13 *Aetocheyletia* individuals were found; 11 were used for taxonomic studies, while two individual mites were used exclusively for molecular analysis. The exoskeletons of these two samples could not be retrieved, as they were destroyed during the isolation process. All collected specimens of *Aetocheyletia* were adult females, no males or immature stages were present in the examined samples. Of the examined feathers, six contained *Aetocheyletia*, five contained other mite taxa but not *Aetocheyletia*. Among the six feathers containing *Aetocheyletia*, five also contained syringophilid mites. The specimens included in this study represent all material of the taxon currently available to the authors.

### 2.2. Light Microscopy and Specimen Preparation

Mites intended for light microscopy were preserved in 75% ethanol. Prior to mounting, specimens were cleared in Nesbitt’s solution for 48 h at room temperature. Cleared specimens were mounted in Berlese medium. Morphological observations were conducted using an Olympus BH2 microscope equipped with differential interference contrast (DIC) optics and a camera lucida for drawings. All measurements are given in micrometres (µm).

### 2.3. Scanning Electron Microscopy (SEM)

Scanning electron microscopy was conducted at the NanoBioMedical Centre of AMU. Specimens prepared for scanning electron microscopy (SEM) were dehydrated in a graded ethanol series up to 99.8%. Drying was carried out using hexamethyldisilazane (HMDS), with specimens immersed in a 1:1 mixture of ethanol and HMDS for 1 min, followed by 100% HMDS for 1 min, and then air-dried under a fume hood. Dried specimens were mounted on carbon adhesive tabs attached to aluminium stubs and sputter-coated with a 10 nm thick layer of gold (Quorum Q150T ES, Laughton, East Sussex, UK). Observations were performed using a JEOL JSM-7001F (Akishima, Tokyo, Japan), Japan scanning electron microscope at an accelerating voltage of 5 kV. Specimens examined using SEM exhibited partial deformation due to dehydration and preparation procedures; therefore, SEM images were used primarily to document surface structures.

### 2.4. Molecular Analysis

Total genomic DNA from two individual mites recovered from quill feathers of *Haliaeetus albicilla* was isolated using the DNeasy Blood & Tissue Kit (Qiagen GmbH, Hilden, Germany) as described by Dabert et al. (2008) [11]. The cytochrome oxidase subunit (COI) gene fragment from the mitochondrial genome region was amplified using PCR with degenerate primers: bcdF01 (CATTTTCHACTAAYCATAARGATATTGG) [12] and bcdR04 (TATAAACYTCDGGATGNCCAAAAAA) [11]. PCR amplifications were performed in a 10 µL reaction volume containing 5 µL of HOT FIREPol^®^ DNA Polymerase (Solis BioDyne, Tartu, Estonia), 0.5 µL of each primer (10 µM), and 4 µL of DNA template. The thermocycling profile was as follows: initial denaturation at 95 °C for 12 min, followed by 40 cycles of 95 °C for 15 s, 50 °C for 1 min, and 72 °C for 1 min, with a final elongation step at 72 °C for 5 min. Following amplification, the PCR products were diluted 1:1 with water, and 5 µL of each sample was separated by electrophoresis on a 1.0% agarose gel. Because of the extremely limited amount of material, additional specimens were not subjected to destructive extraction to avoid compromising the morphological type series.

### 2.5. Terminology

Terminology for gnathosomal setation follows Grandjean [13]. Idiosomal chaetotaxy follows Grandjean [14] as adapted for Prostigmata by Kethley [15]. Leg chaetotaxy is based on the system proposed by Grandjean [16] and further developed by Kethley [17] and Bochkov [18]. Length of setae for the holotype is given before the brackets, while the values inside the brackets represent the range observed in the paratypes.

### 2.6. Deposition of Material

The holotype and paratype specimens of *Aetocheyletia variata* sp. n. are deposited in the acarological collection of Adam Mickiewicz University, Poznań, Poland (AMU), under accession number EG (25–1101) 001–002.01–12.

Additional mite taxa collected from the same material are deposited under accession numbers EG (25–1101) 001–002.01–35 (*Megasyringophilus* sp.) and EG (25–1101) 001–002.01–39 (*Peristerophila* sp.).

### 2.7. Ethical Statement

The study was conducted on feathers collected from deceased birds found in the field. No live animals were used; therefore, ethical approval was not required. In Polish law, holding and collecting feathers of strictly protected species requires no supplementary authorization, according to the Regulation of the Minister of the Environment of 16 December 2016 on the Protection of Animal Species, § 9, item 7 (Dz. U. 2022, poz. 2380).

## 3. Results

### 3.1. Systematic

Family: Cheyletidae

Tribe: Metacheyletinii

#### 3.1.1. Diagnosis

##### Genus: *Aetocheyletia* gen. n.

Larviform and oval shape, medium size mites, total body length 790–855 µm long. *Gnathosoma* 125–140 µm long. Hypostomal apex without protuberances, bearing adoral setae *ao1*, *ao2*. Subcapitulum densely punctate, subcapitular setae *n* present. The medial branches of the peritremes flattened, and both the medial and lateral branches with numerous minute chambers. Palpal well-developed ca. 125 µm long, densely punctate. Palpal claw bearing one well-developed dorsal tooth and one additional minute tooth on the ventral side. Palpal chaetotaxy: femur with *dF*; palpal with *dG*; tibia with *dTi*, *l*’*Ti* ventrally, *l*”*Ti* dorsally; palpal tarsus—smooth eupathidia *acm*, *sul*, *ul*’, *ul*”*,* and single solenidion ω, setae *elcp* small and lumpy-like. *Idiosoma*. Propodonotal shield elliptic shape, with indiscernible anterior margin. Bases of setae *c2* situated on ventral side, located at the same transverse level as setae *se*. Setae *d2* and *f1* absent. Pseudanal setae represented three pairs of setae (*ps1*-*3*) and two pairs of the genital setae (*g1* and *g2*). *Legs*. Legs I–III present, legs IV completely absent, cuticular vestiges of legs IV present. Coxal fields I–III sclerotized and punctate. Setae *4a* absent. Leg I–III setation: I—coxal field bearing bases of setae *1b*, trochanter without of setae, femur with setae *d* and *v*”, genu with one setae *l*” and solenidion (σ) dorsally, tibia with 4 setae *d*, *l*”, *v*’, *v*” and single solenidion (φ), tarsus with 8 setae *ft*, *tc*’*, tc*”*,* single solenidion (ɷ), *p*’, *p*”, *u*’, *u*”, *vs*, legs II–III—femur with *d* and *v*”, genu with *l*’, tibia with 4 setae *d*, *l*’, *v*’, *l*”, tarsus with 5 setae *a*’, *tc*’, *tc*”, *u*’, *u*”.

##### Habitat

Body feathers and tail coverts.

##### Type Host

*Haliaeetus albicilla* (Linnaeus, 1758) (Accipitriformes: Accipitridae).

##### Type Species

*Aetocheyletia variata* sp. n.

##### Differential Diagnosis

*Aetocheyletia* gen. n. is morphologically most similar to *Metacheyletia* Fain, 1972. Females of both genera have an oval, larviform body, three pairs of functional legs, complete reduction in leg IV, and three and two pairs of pseudanal and genital setae, respectively. *Aetocheyletia* differs from *Metacheyletia* by a stable combination of two qualitative gnathosomal characters: the presence of subcapitular setae *n* and an additional minute ventral tooth on the palpal claw. Both characters were consistently observed in all examined females of *A*. *variata*. In contrast, subcapitular setae *n* and the additional ventral tooth are absent from the available diagnoses and sufficiently detailed descriptions of the currently recognized species of *Metacheyletia*. The difference in subcapitular chaetotaxy concerns the presence or absence of a homologous setal element rather than variation in setal length or proportion, which is conventionally used to distinguish species within *Metacheyletia*. A detailed comparison with the recognized species of *Metacheyletia* is provided in Table S1.

Known host association*. Aetocheyletia* is currently known only from *Haliaeetus albicilla* (Accipitriformes: Accipitridae), whereas species of *Metacheyletia* have been recorded from Psittaciformes, Passeriformes, Coraciiformes, and Musophagiformes.

##### Etymology

This generic name derives from the Greek “aetos”, meaning eagle, and refers to the host association with birds of prey.

#### 3.1.2. Description

##### Aetocheyletia variata sp. n.

**Figure 1:**
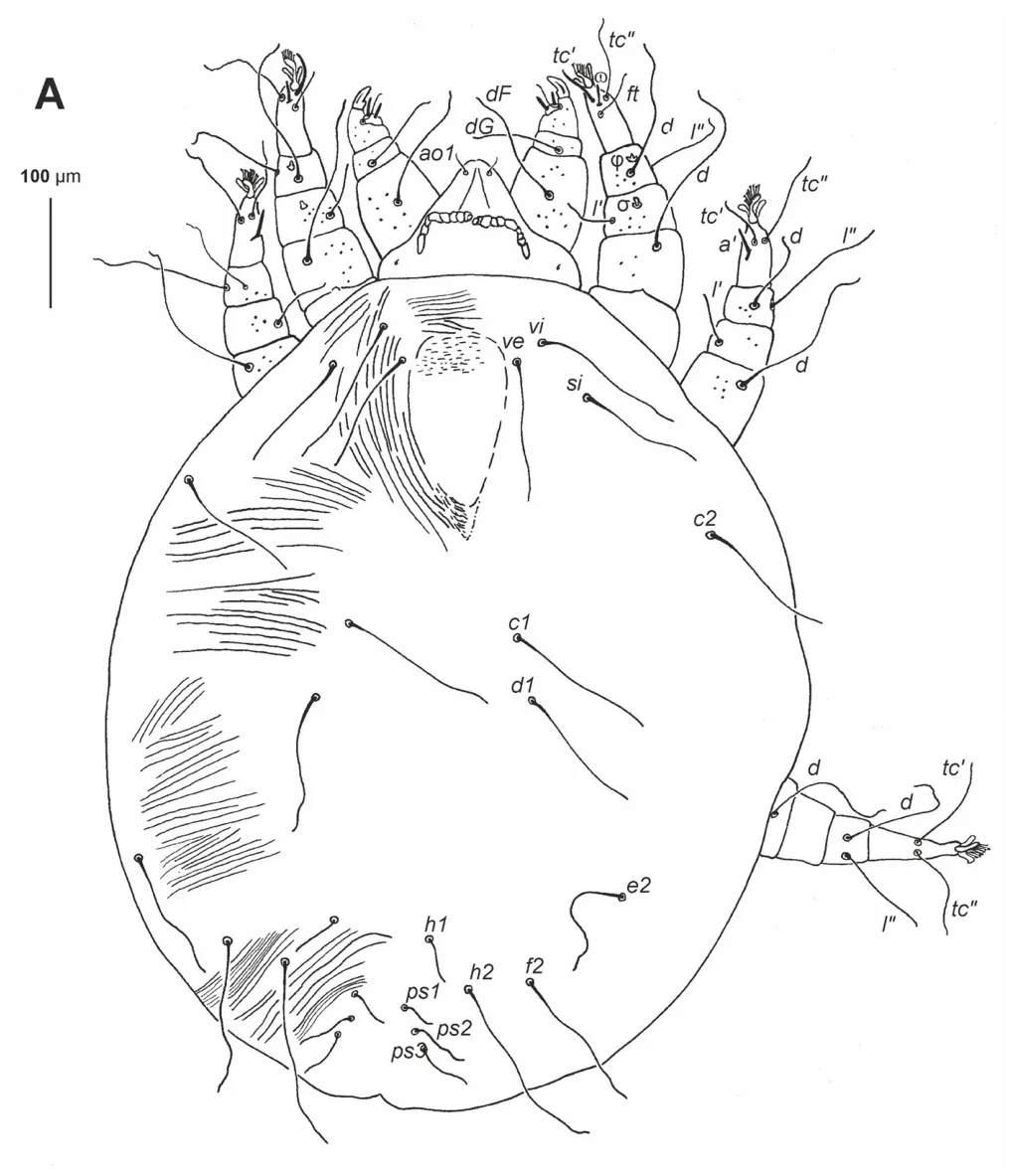

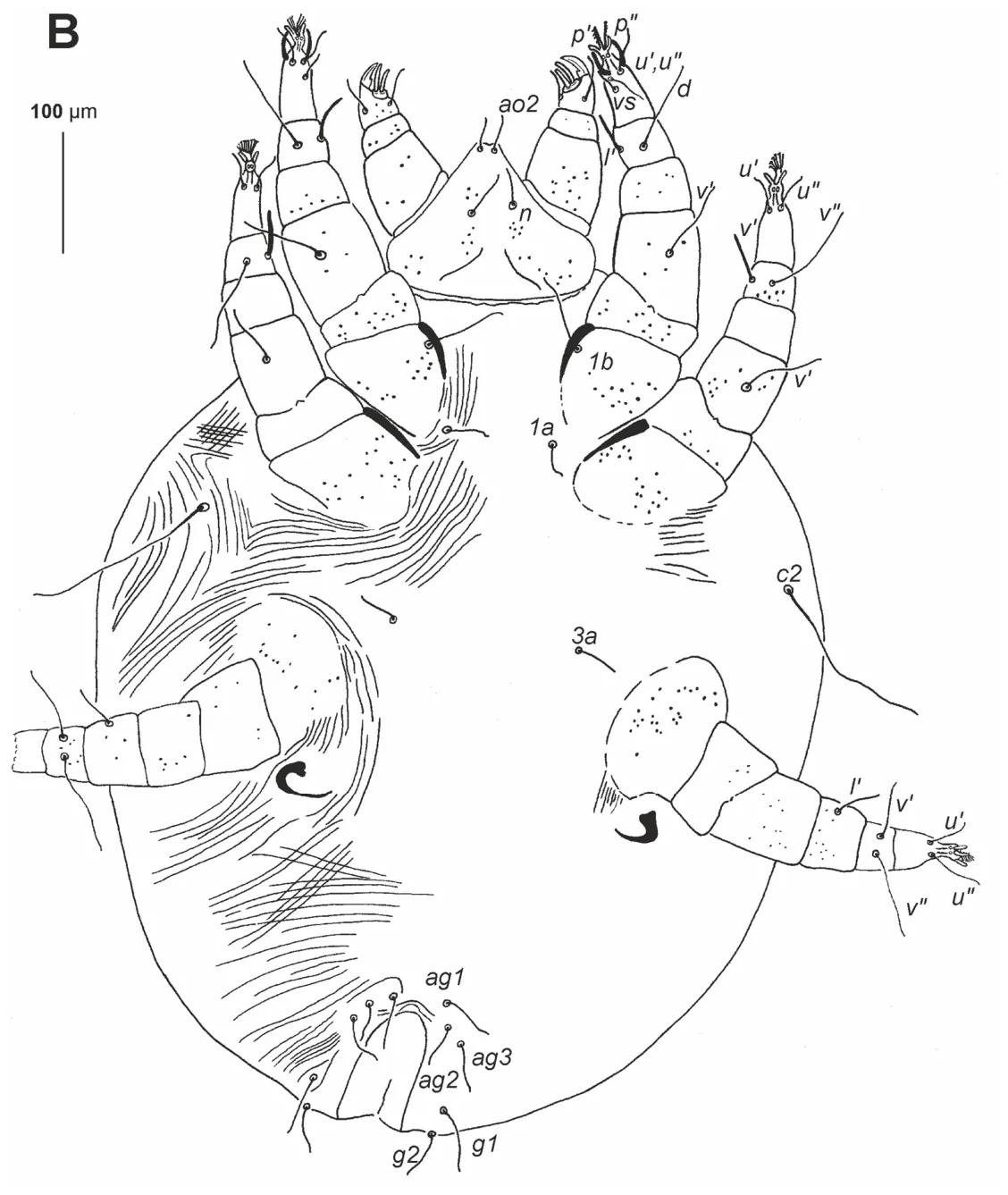
(**A**) *Aetocheyletia variata* sp. n. holotype female, dorsal view. (**B**) *Aetocheyletia variata* sp. n. holotype female, ventral view.

**Figure 2:**
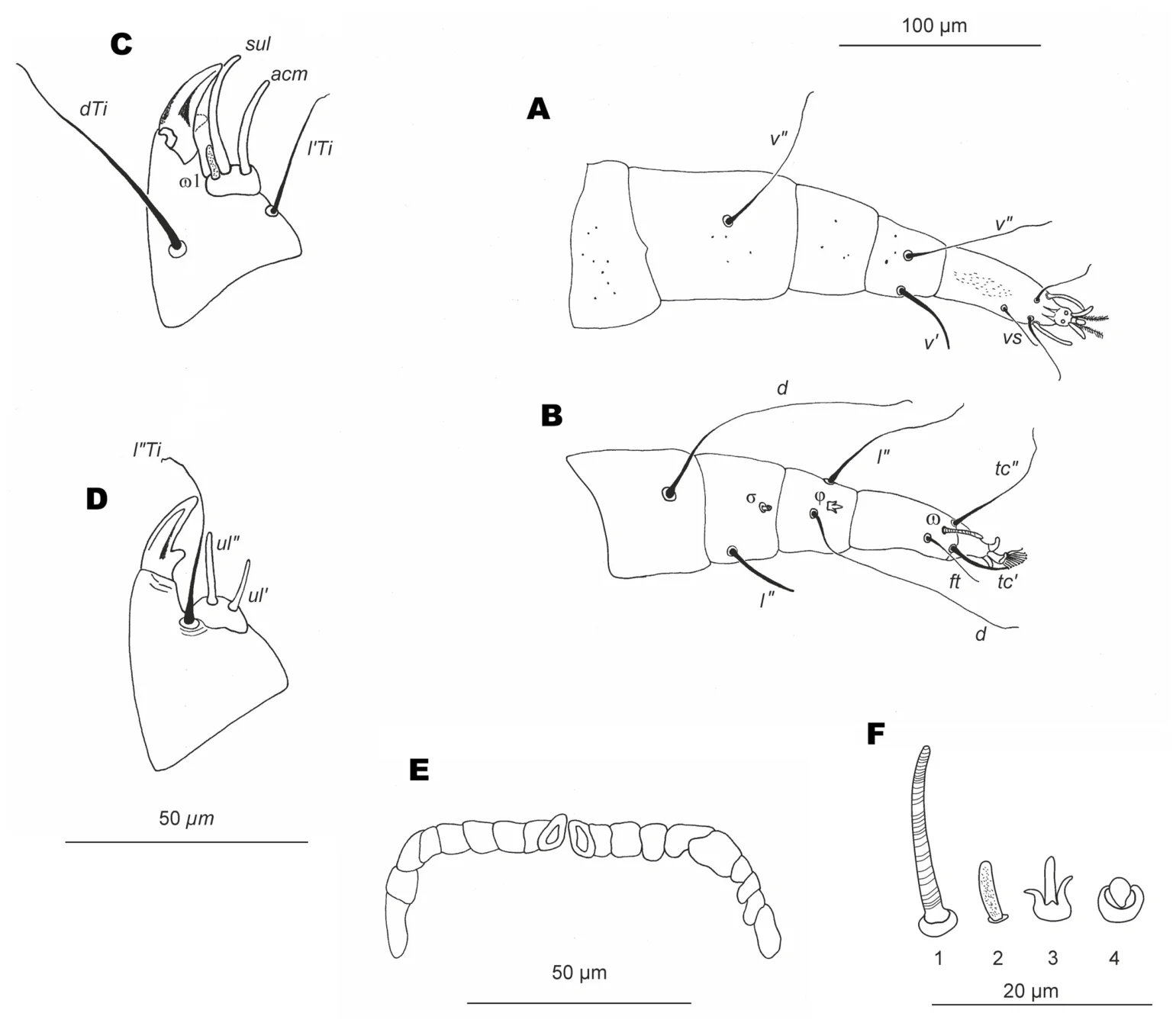
*A*. *variata* details of holotype female. (**A**) Leg I (left) in ventral view. (**B**) Same in dorsal view. (**C**) Palpal tibio-tarsus in ventral view. (**D**) Same in dorsal view. (**E**) Peritremal branch. (**F**) Solenidions: ɷ1—[1] on tarsus I, [2] on palpal tibio-tarsus, φ—[3] on tibia leg I, σ—[4] on genu leg I.

**Figure 3:**
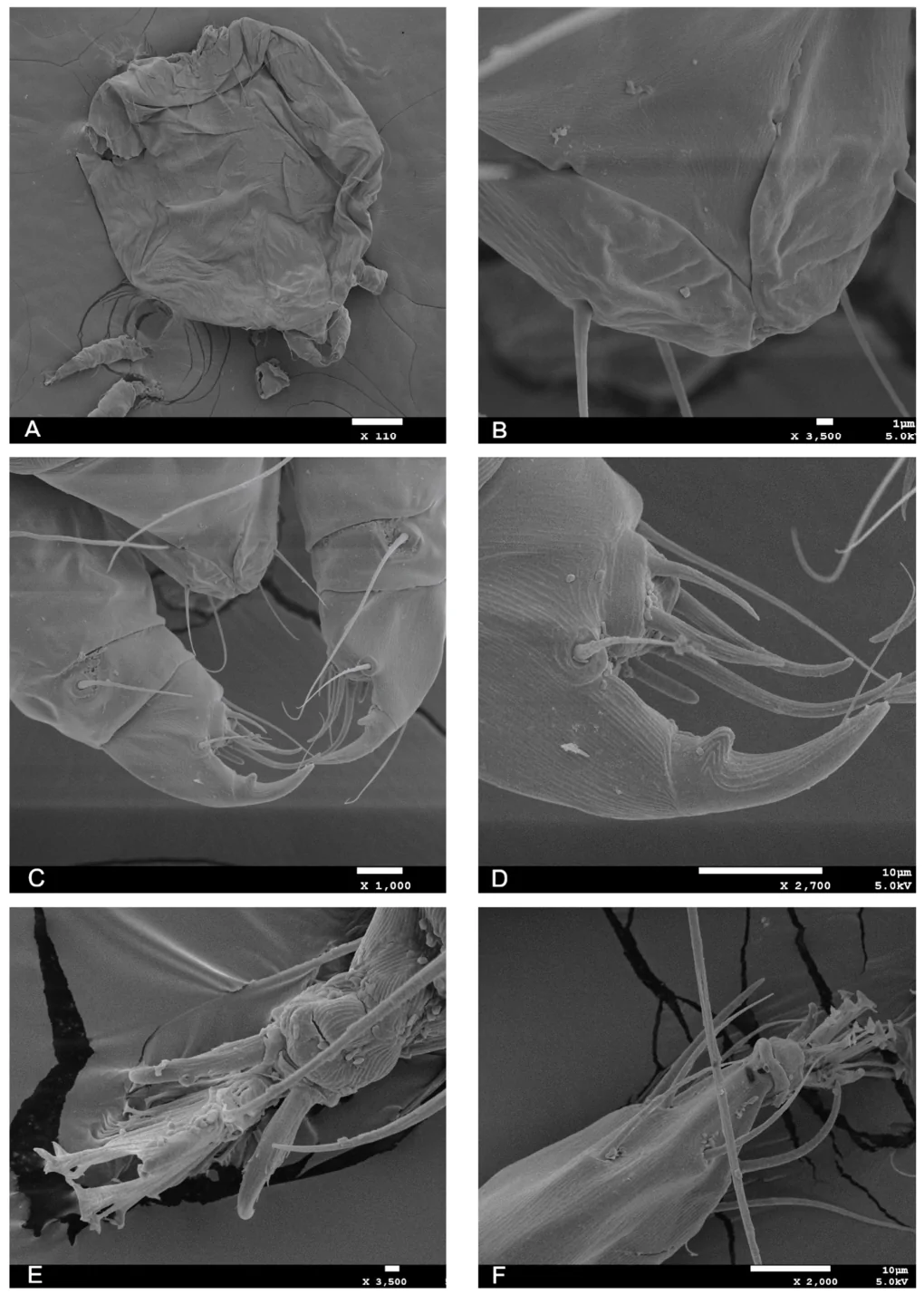
SEM micrographs. Dorsal view of the female’s body. [Specimens prepared for SEM were partially deformed due to dehydration and drying, therefore, SEM images were used primarily to document surface structures] SEM micrographs. Details of female *A*. *variata*. (**A**) Female dorsal view. (**B**) Dorsal aspect of the hypostomal apex. (**C**) Dorsal view of the gnathosoma. (**D**) Dorsal view of the palpal tarsus with visible claw and tooth. (**E**) Dorsal view of the tarsus I. (**F**) The same as the ventral aspect.

Female holotype (Figure 1A,B). Total body length 790 (840–855 in 10 paratypes). *Gnathosoma* 130 (125–140) µm long. Hypostomal apex truncate. Subcapitulum densely punctate, pair of subcapitular setae *n* present and measured 35–50 µm. Each medial branch of peritremes with 5–6 chambers, each lateral branch with 4 chambers. Idiosoma. Propodonotal shield elliptic shape with visible granulation on anterior margin. Setae *vi*, *ve*, *si* similar in length (110–140 µm long) and situated outside propodonotal shield. Coxal fields I–III densely punctate, cuticular vestiges of legs IV present. Legs I–III variable punctate, legs setae filiform or rod-shaped (Figure 2 and Figure 3). Lengths of setae: *vi* 130 (125–135), *ve* 110 (110–140), *si* (110–130), *se* 120 (120–145), *c1* 130 (110–135), *c2* 155 (125–155), *d1* 120 (110–130), *e2* 120 (110–130), *f2* 110 (110–145), *h1* (130–155), *h2* 140 (140–180), *ps1*–*ps3* 25–35, *g1* and *g2* 35–55, *ag1* 45 (30–35), *ag2* 40 (30–45), *ag3* 55 (35–45), *1a* 35 (30–45), *1b* (55–85), *3a* (40–60).

##### Type Material

Female holotype and four female paratypes from quill of body feathers and tail coverts of White-Tailed Eagle *Haliaeetus albicilla* (Linnaeus, 1758) (Accipitriformes: Accipitridae), Poland: Wielkopolska, Karwin, Lubuskie, 26 December 2023 coll. Mizera and six females paratypes from quill of juvenile tail covert, Biały Bór, Zachodniopomorskie Voivodeship, Poland, 12 August 2024 coll. Ignacy Gołębiewski.

##### Type Material Depositions

Female holotype and 10 females paratypes are deposited in the AMU, EG (25–1101) 001-002.01-12.

##### Etymology

The species name *variata* (Latin: “variable”) refers to the pronounced variability in the number and arrangement of setae observed in females.

*Remarks*. Two distinct mite groups belonging to the superfamily Cheyletoidea were found co-occurring within the same quill feather: *Aetocheyletia* (Cheyletidae) and mites of the genera *Megasyringophilus* and *Peristerophila* (Syringophilidae). Co-occurrence of all taxa within the same quill was not observed.

##### Molecular Analysis

PCR amplification of the mitochondrial cytochrome c oxidase subunit I (COI) gene fragment was attempted for seven individual mites. No PCR products of the expected size were detected by agarose gel electrophoresis in any of the analyzed specimens. Consequently, sequencing was not performed, and no molecular sequences were obtained or deposited in a public database.

### 3.2. Variability of Chaetotaxy in Females of Aetocheyletia

**Figure 4:**
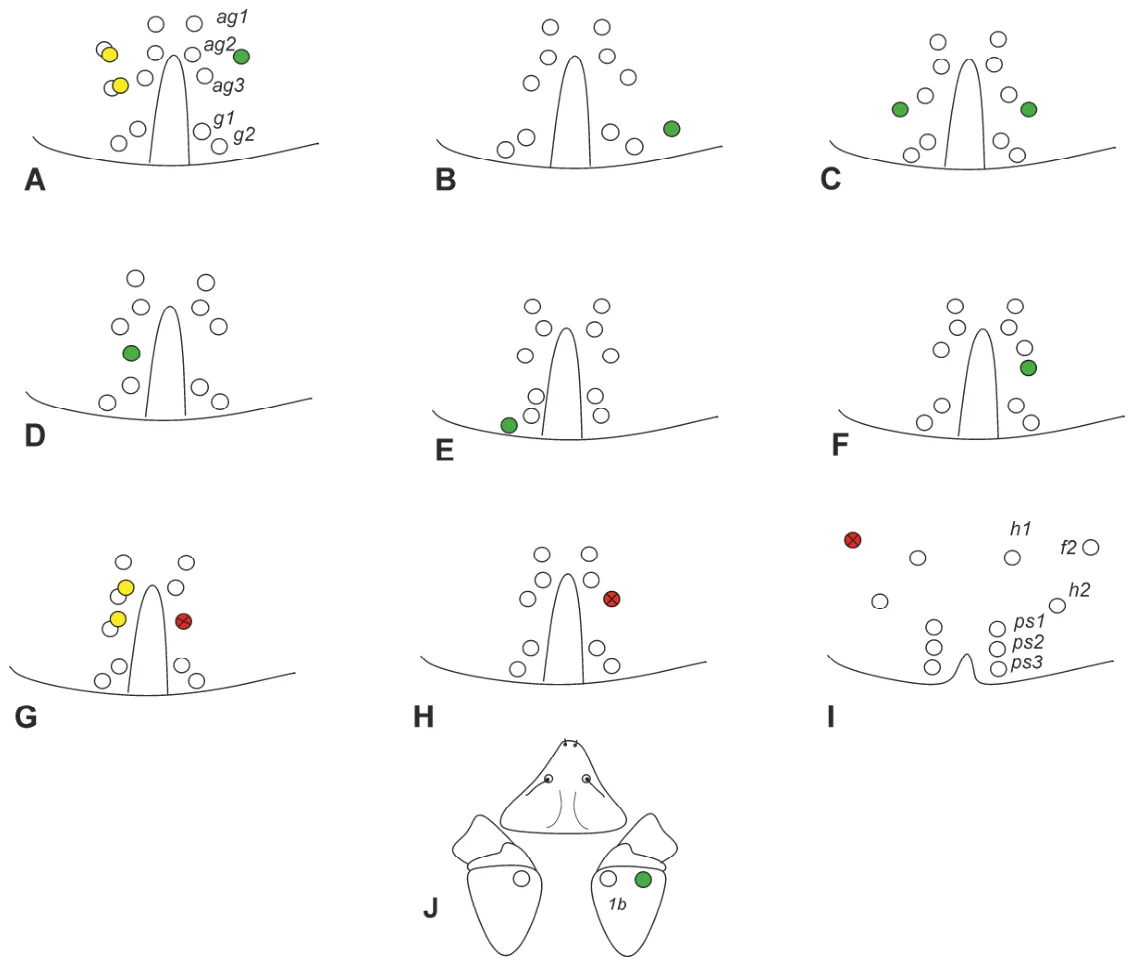
Schematic representation of localized idiosomal setal anomalies in females of *Aetocheyletia variata*.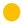 duplicated setae (**A**,**G**); 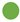 supernumerary setae (**A**–**F**,**J**); 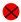 setal reduction (**G**–**I**).

Morphological and chaetotactic analyses revealed localized quantitative anomalies in idiosomal setation. Only one of the 11 examined females exhibited the standard idiosomal setal complement and arrangement illustrated in Figure 1A,B. In the remaining 10 specimens, one or more localized anomalies were recorded, predominantly in the aggenital and genito-anal regions. These anomalies included setal duplication, supernumerary setae, and setal reduction and did not constitute a consistent alternative chaetotactic pattern (Figure 4). The most frequent anomalies occurred in the aggenital and genito-anal regions. In the majority of cases, the predominant anomaly (observed in seven specimens) was the presence of additional setae. The bases of these supernumerary setae were usually situated near the bases of aggenital (*ag*) setae or between those of neighboring idiosomal setae. Symmetrically arranged pairs of additional setae were also observed close to *ag3*. Additional setae occurred near the bases of setae *g2* and on coxal I near setae *1b* on the right side of the body (Figure 4A–F,J). Duplication of setae was recorded in two cases, where paired setae originated from the same alveolus at the positions corresponding to *ag2* and *ag3* (Figure 4A,G). Setal reduction was less frequent and was observed in three specimens, involving the absence of *ag3* on the right side and *h2* on the left side of the body. In several individuals, asymmetry in setal distribution between the left and right sides of the body was observed (Figure 4G–I). No anomalies involving duplication, reduction, or additional setae were detected in the propodonotal region.

Importantly, subcapitular setae *n* and the additional minute ventral tooth on the palpal claw were consistently present in all examined females. No absence, asymmetry, or other detectable variation was observed in either diagnostic character. Thus, the recorded anomalies affected localized idiosomal setal positions and did not involve the characters used in the generic diagnosis. Because all available specimens were adult females, the present analysis assesses variation only within this sex and developmental stage and does not address sexual or ontogenetic variation. Nevertheless, the principal diagnostic characters, namely the subcapitular setae *n* and the additional minute ventral tooth on the palpal claw, were consistently present in all examined specimens.

### 3.3. The Quill Wall Deformation Associated with Infestation by A. variata

**Figure 5:**
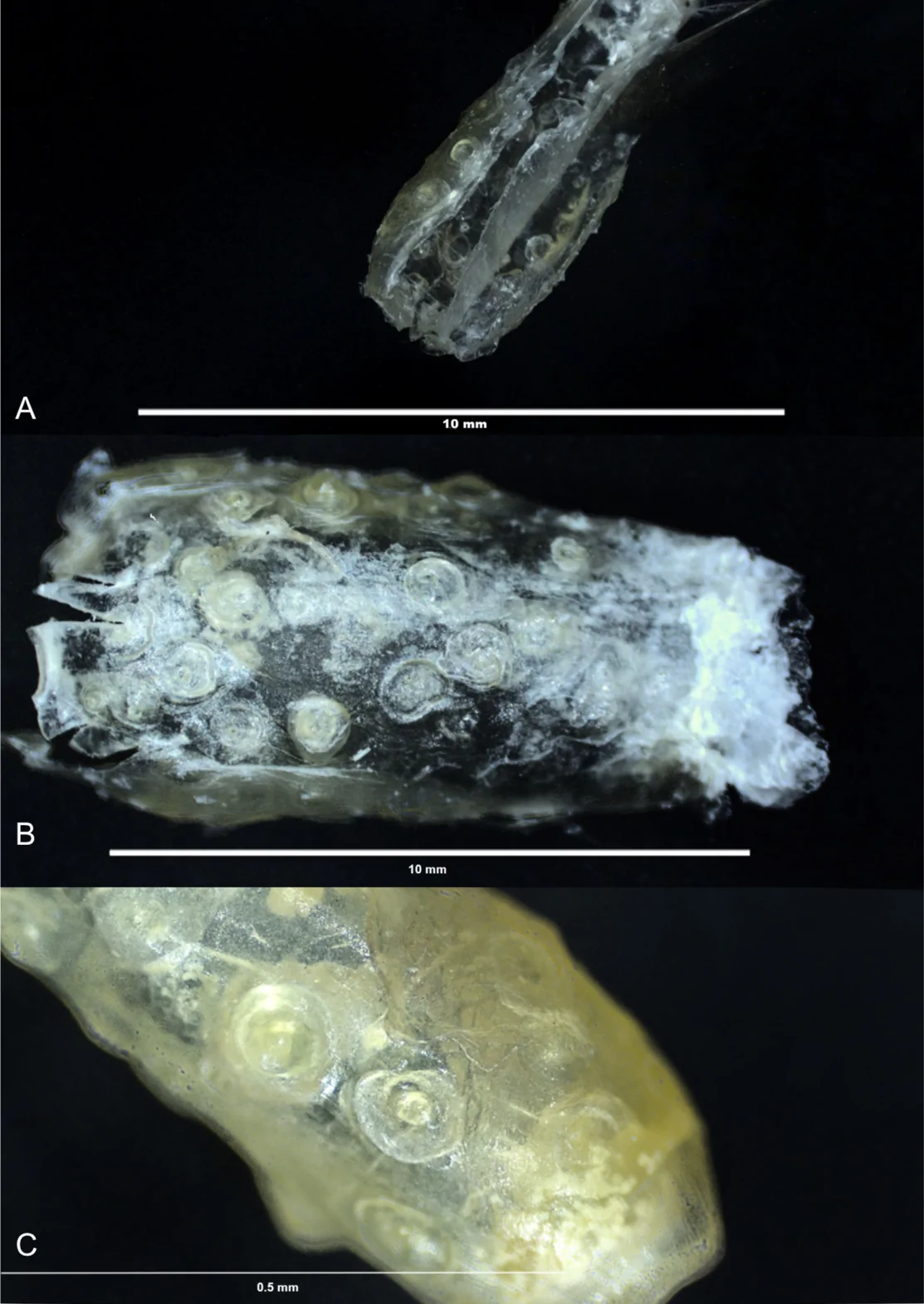
Quill-wall deformations associated with infestation by *A*. *variata*. (**A**) The quill wall with numerous spiral deformations situated within the external layer. (**B**,**C**) Variation in the number of circular structures on different feathers, appearing as distinct, apex-like bulges.

In addition, conspicuous deformations of the external surface of quills infested by *Aetochyletia* were recorded. In total, 11 feathers were infected by mites belonging to two different families: Syringophilidae (*Megasyringophilus* and *Peristerophila*) and Cheyletidae (*Aetocheyletia*). *A*. *variata* was detected in six feathers, and all of them exhibited quill-wall deformations. Among the five feathers in which *Aetocheyletia* was not detected, no comparable deformations were observed (Table 1). These deformations were round in outline and resembled spiral channels (‘club-quill’) with a slightly pointed apex. The bulges were numerous and irregularly distributed along the quill shaft. Internally, the quill wall surrounding the bulging areas was smooth and intact (Figure 5A–C). However, the external wall was split, forming an additional medial layer. The exclusive occurrence of these structures in infested quills is consistent with a possible role of *Aetocheyletia* in the formation of these deformations. This distribution demonstrates an association within the examined material but does not establish causality.

## 4. Discussion

Mites of the tribe Metacheyletinii Fain, 1980 remain a poorly recognized and taxonomically narrow lineage within the family Cheyletidae, previously represented exclusively by the genus *Metacheyletia* Fain, 1972 [4]. The establishment of *Aetocheyletia variata* gen. n., sp. n. therefore, constitutes both a taxonomic expansion of the tribe and a significant extension of its known host range to Accipitriformes.

Morphologically, *Aetocheyletia* conforms to the strongly regressed, larviform body plan characteristic of Metacheyletinii, including reduction of idiosomal chaetotaxy and the retention of only three functional pairs of legs [8,9]. Such features are generally interpreted as adaptations to the confined and relatively stable environment of the feather quill [1].

The separation of *Aetocheyletia* from *Metacheyletia* is based on a stable combination of two qualitative gnathosomal characters: the presence of subcapitular setae *n* and an additional minute ventral tooth on the palpal claw. The first character represents the presence of a homologous element of subcapitular chaetotaxy, whereas the second represents an independent structural modification of the palpal claw. Both characters were stable in all examined females. Both characters were consistently present in all examined specimens of *A*. *variata*. In contrast, subcapitular setae *n* are absent from the available diagnoses and sufficiently detailed descriptions of all currently recognized species of *Metacheyletia*, whose palpal claw also lacks the additional ventral tooth. This combination differs from the characters conventionally used to distinguish species of *Metacheyletia*, which are predominantly quantitative and include setal lengths, proportions, and length ratios.

In the available key to *Metacheyletia* [7] the presence or absence of seta *ag3* is the only distinction involving setal number, whereas the presence or absence of remnants of legs IV represents an additional qualitative, but non-chaetotactic, character. Variation in *ag3* should not, however, be regarded as directly equivalent to the condition of subcapitular setae *n*. Seta *ag3* is located in the aggenital region, where additions, reductions, duplications, and asymmetries were recorded in the present material. In contrast, no variation was observed in the subcapitular setae or in the structure of the palpal claw. Thus, the proposed genus is supported not by the presence of a single seta alone, but by a stable combination of two qualitative gnathosomal characters. The recognition of *Aetocheyletia* is therefore presented as a morphology-based taxonomic hypothesis supported by the available comparative evidence and open to reassessment when more comprehensive comparative material becomes available.

Knowledge of prostigmatic mites inhabiting birds of prey remains limited. The superfamily Cheyletoidea comprises three families permanently associated with birds: Cheyletidae, Harpirhynchidae and Syringophilidae [4]. However, to date, representatives of only two families, Harpirhynchidae and Syringophilidae, have been documented from Accipitriformes. The discovery of *A*. *variata* in a new host group therefore suggests that the diversity of Metacheyletinii is likely much higher than currently recognized, particularly among poorly sampled avian lineages such as birds of prey. The occurrence of Metacheyletinii in a bird of prey host is particularly noteworthy. Previous records of the tribe were restricted mainly to passerines and parrots, with isolated reports from several additional avian orders [6,7,8,9,10]. This distribution pattern likely reflects uneven sampling rather than strict host specificity [1]. Birds of prey remain comparatively poorly investigated with respect to their acarofauna, and the present discovery suggests that additional, undescribed diversity of quill-inhabiting cheyletids may occur within these hosts.

The available material represents two independent records from a single host species and consists exclusively of adult females. These limitations prevent an assessment of sexual and ontogenetic variation and do not permit firm conclusions regarding the geographic distribution or host specificity of the taxon. In studies of rare, permanently parasitic mites, however, the composition of the available material is determined largely by the exceptional and unpredictable occurrence of the parasite rather than by an ideal sampling design.

To our knowledge, this taxon has remained unrecorded despite many years of extensive research by our group and other specialists on mites permanently associated with birds, encompassing material from hundreds of avian species and resulting in the discovery and description of numerous new quill-mite and other avian-associated mite taxa. Its recovery from only two localities and a single host species should therefore be considered in the context of this broader sampling effort. Nevertheless, because the sampling was not designed to estimate prevalence or host specificity, the available records do not allow us to determine whether the taxon is genuinely rare, has a restricted host association, or has simply remained undetected.

Molecular analyses were attempted but did not yield usable sequences. This outcome may have resulted from the very small quantity of starting material and possible DNA degradation associated with the condition and preservation of the host material. Further destructive extraction would have required sacrificing a substantial proportion of the available type series. In addition, no molecular sequences are currently available for any recognized species of *Metacheyletia*. Consequently, even an isolated sequence of *Aetocheyletia* would not presently permit a direct molecular test of the proposed distinction between the two genera. The available morphological data also cannot exclude cryptic diversity conclusively, although no consistent alternative morphological groups were identified among the examined females. The generic assignment should therefore be regarded as a testable morphology-based hypothesis that requires reassessment when suitably preserved specimens and comparative molecular data from *Metacheyletia* become available.

For rarely encountered, permanently parasitic mites, it is often not possible to obtain both sexes, all developmental stages, and material from multiple hosts and localities simultaneously. Males and immature stages were not selectively excluded from the present study but were absent from the collected samples. Although additional material is required for a complete evaluation of sexual, ontogenetic, geographic, and host-associated variation, postponing the description until an ideally structured sample becomes available could leave this morphologically distinctive taxon undocumented indefinitely.

Members of Metacheyletinii differ substantially from the typical quill-mite morphotype, which is usually characterized by an elongated body, long filiform setae, and weak sclerotization [2,18]. In contrast, *Metacheyletia* and *Aetocheyletia* possess compact, oval bodies with shortened setae and a reduced gnathosoma equipped with short cheliceral stylets. Similar reductions are known in other highly specialized quill-inhabiting mites, including representatives of the family Apionacaridae Gaud et Atyeo, 1977 [19]. In these taxa, simplification of chaetotaxy and body organization has previously been interpreted as an adaptation to the spatially restricted quill environment [9,18,20]. Paedomorphosis, understood as the retention of juvenile-like characters in adult stages [21], has also been proposed as an explanation for the reduced, larviform morphology of Metacheyletinii. However, the present study includes no ontogenetic or comparative phylogenetic data that would allow these interpretations to be tested in *Aetocheyletia*. Comparisons with other quill-inhabiting mites therefore provide a relevant morphological context, but do not demonstrate that the observed reduction resulted from paedomorphosis, convergence, or adaptation to the quill environment.

A notable feature of *A*. *variata* is the occurrence of localized anomalies in idiosomal chaetotaxy. These included duplication, reduction, and addition of setae, sometimes accompanied by asymmetry between the two sides of the body. The position and form of the anomalies differed among specimens and did not constitute a consistent alternative chaetotactic pattern. Most anomalies were concentrated in the aggenital and genito-anal regions, although isolated changes were also observed at other idiosomal setal positions. Similar anomalies have been reported in other mite groups and have been discussed in relation to developmental instability, fluctuating asymmetry, evolutionary reduction, or environmental effects [22,23,24]. The present material, however, does not permit these possible mechanisms to be distinguished. We therefore interpret the observed features conservatively as localized chaetotactic anomalies of unknown origin rather than as evidence of a specific developmental or evolutionary process. Importantly, these anomalies did not involve the characters supporting the generic diagnosis. Subcapitular setae *n* and the minute ventral tooth on the palpal claw are located in anatomically separate gnathosomal structures and were consistently present in all examined females. Localized variability in idiosomal setation therefore does not, by itself, demonstrate instability of these diagnostic structures. The reduced, larviform morphology of *Aetocheyletia* resembles that previously described in *Metacheyletia*, but its developmental and evolutionary origin remains unresolved. Additional specimens, developmental stages, and comparative data from related taxa will be required to evaluate the processes underlying both the structural reduction and the observed chaetotactic variability. Nevertheless, the available material cannot exclude the possibility that additional variation may be detected in other populations or developmental stages. The stability of the diagnostic characters should therefore be reassessed when further material becomes available.

Another noteworthy observation is the repeated co-occurrence of *Aetocheyletia* and syringophilid mites within the same feather quills. Co-habitation of a single quill by mites belonging to different families appears to be relatively uncommon and has most often been documented when parasitic and predatory taxa occupy the same microhabitat. Comparable associations include members of the tribe Cheletosomatini, which comprise predators inhabiting feather quills and preying upon parasitic astigmatic mites and syringophilids [1]. Previous authors suggested that species of *Metacheyletia* may exploit perforations produced by syringophilid mites and may therefore benefit from their presence within the quill microhabitat [3]. In the present material, syringophilids occurred in five of the six feathers containing *Aetocheyletia*. This frequent co-occurrence is noteworthy, but does not provide direct evidence that *Aetocheyletia* depends on, or is facilitated by, syringophilid mites. Observations of red-stained intestinal contents in *A*. *variata* are consistent with the ingestion of host-derived material, although the precise feeding mechanism remains unknown. Feather quills represent spatially restricted and structurally heterogeneous microhabitats, and different mite groups may occupy different regions or use different resources within the same feather. The observed co-occurrence may therefore reflect shared habitat requirements, independent exploitation of the quill environment, or an ecological interaction that cannot be identified from the available material. The present observations do not demonstrate facilitation, dependence, predation, competition, or another direct relationship between the taxa. Resolving the ecological significance of this association will require direct observations of living mites and broader comparative sampling.

In addition, we documented previously unreported circular and spiral deformations of the quill wall. Within the examined material, these structures occurred exclusively in feathers containing *Aetocheyletia*. Other feathers collected from the same host individual, including those inhabited by syringophilid mites, showed no comparable alterations. Within the examined material, quill-wall deformations occurred in six feathers containing *Aetocheyletia variata*, whereas no comparable alterations were detected in five feathers without this taxon, including feathers containing other mites. Furthermore, comparable quill-wall deformations have not been observed during the authors’ extensive studies of Syringophilidae from numerous avian hosts. These observations indicate an association between the presence of *Aetocheyletia* and the quill-wall deformations, but do not demonstrate that the mites caused them. Several explanations remain possible. The deformations could result from direct or indirect effects of mite activity, developmental abnormalities of the feather, previous mechanical injury, microbial or fungal processes, host-related pathological changes, or another unidentified factor associated with the affected quills. The present study was based on fully developed feathers collected from deceased birds and therefore did not permit temporal observation of deformation development. Histological examination of the affected feather tissues was also not performed. Consequently, it remains unknown whether the observed structures developed before, during, or independently of infestation by *Aetocheyletia*. Although causality cannot presently be established, the exclusive occurrence of these distinctive structures in the examined quills containing *Aetocheyletia*, together with their absence from feathers containing only other mite taxa, provides a basis for a testable hypothesis. Determining their origin will require histological investigation, examination of freshly collected material, broader comparative sampling, and, where feasible, temporal or experimental observations.

Taken together, the present observations expand current knowledge of the morphology, host associations, and poorly understood biology of Metacheyletinii. The study documents a strongly reduced, larviform morphology, frequent but localized chaetotactic anomalies, repeated co-occurrence with syringophilid mites, and an association between the presence of *Aetocheyletia* and distinctive quill-wall deformations within the examined material. However, the available data do not resolve the developmental or evolutionary origin of the reduced morphology, the causes of the chaetotactic anomalies, the ecological significance of co-occurrence with syringophilids, or the origin of the quill-wall deformations. Future studies incorporating additional specimens and developmental stages, broader host sampling, comparative molecular data, direct observations of living mites, and histological examination of affected feathers will be necessary to evaluate these questions.

## 5. Conclusions

The present study establishes *Aetocheyletia* gen. n., with the type species *A*. *variata* sp. n., within the tribe Metacheyletinii and represents the first record of this tribe from birds of prey and from the order Accipitriformes. Recognition of the new genus is supported by a stable combination of qualitative gnathosomal characters distinguishing it from *Metacheyletia*.

This finding expands the known host range of Metacheyletinii and suggests that the diversity and host associations of this poorly studied group may remain underestimated. The examined females exhibited frequent but predominantly localized anomalies in the number and arrangement of idiosomal setae. These anomalies did not affect the subcapitular setae *n* or the additional minute ventral tooth on the palpal claw, which were consistently present in all morphologically examined specimens and constitute the principal characters supporting the generic diagnosis.

Syringophilid mites co-occurred with *Aetocheyletia* in five of the six feathers containing the new taxon. This observation documents the use of the same quill material by mites belonging to different families, but does not establish facilitation, dependence, resource partitioning, or another direct ecological interaction between them. Similarly, quill-wall deformations were recorded in all six feathers containing *Aetocheyletia*, whereas no comparable alterations were observed in five mite-positive feathers without this taxon. This distribution supports an association within the examined material, but does not demonstrate that the mites caused the observed deformations. The present diagnosis is necessarily restricted to adult females collected from *Haliaeetus albicilla* at two localities. The proposed classification should therefore be reassessed when males, immature stages, additional populations, host records, and comparative molecular data become available. Histological examination of affected feather tissues and direct observations of living mites will also be necessary to clarify the feeding biology of *Aetocheyletia*, the significance of its co-occurrence with other quill mites, and the origin of the observed quill-wall modifications.

## Figures and Tables

**Table 1:** Distribution and numbers of specimens of *Aetocheyletia variata* and syringophilid mites in the 11 examined mite-infested quill feathers, with the corresponding presence or absence of quill-wall deformations.

Host ID	Feather ID	Feather Type	*Megasyringophilus* sp. (Syringophilidae) No. of Specimens	*Peristerophila* sp. (Syringophilidae) No. of Specimens	*Aetocheyletia variata* (Cheyletidae) No. of Specimens	Quill-Wall Deformation: Present (+)/Absent (−)
HA ALB_22	1	body covert	2	0	0	−
HA ALB_22	2	body covert	2	0	0	−
HA ALB_23	3	juv. tail covert	6	0	2	+
HA ALB_23	4	juv. tail covert	4	0	4	+
HA ALB_22	5	tail covert	21	0	4	+
HA ALB_23	6	juv. tail covert	3	2	0	−
HA ALB_22	7	tail covert	7	0	1	+
HA ALB_22	8	tail covert	0	39	1	+
HA ALB_23	9	tail covert	2	0	0	−
HA ALB_23	10	tail covert	2	0	0	−
HA ALB_22	11	body covert	0	0	1	+

## Data Availability

Data are available upon request from the corresponding authors. This study has been registered in the ZooBank database under LSID number: urn:lsid.org:pub:1B0664A2-9EE5-4D59-B03B-826FE557AACF.

## Supplementary Materials

The following supporting information can be downloaded at: https://www.mdpi.com/article/10.3390/ani16193151/s1, Table S1: Comparative feature analysis of *Aetocheyletia* gen. n. and *Metacheyletia* spp.
