# Supplementary material for: Quill-Dwelling Cheyletid Mites in Birds of Prey: A New Genus from Haliaeetus albicilla and Evidence of Feather-Shaft Modification
Source: Animals (Basel). 2026 Oct 8;16(19):3151. doi: 10.3390/ani16193151 (PMC13663453; doi:10.3390/ani16193151)
Supplement: Supplementary file 1 [file animals-16-03151-s001.zip › animals-4523252-supplementary.pdf]

Character	*Aetocheyletia* gen. n.	*Metacheyletia*
Adult morphology	Highly regressed, larviform	Highly regressed, larviform
Functional legs in adults	Three pairs	Three pairs
Legs IV	Absent/vestigal	Absent
Subcapitular setae *n*	Present	Absent
Ventral tooth on palpal claw	Present	Absent
Pseudanal setae	Three pairs	Three pairs
Genital setae	Two pairs	Two pairs
Host association	Accipitriformes	Psittaciformes, Passeriformes, Coraciiformes, Musophagiformes
Distribution	Poland	Africa, Old and New Worlds

Table S1. Comparative feature analysis of *Aetocheyletia* gen. n. and *Metacheyletia* spp.
